# Case Series of Multisystem Inflammatory Syndrome (MIS-C) in Children during the SARS-CoV-2 Pandemic in Latvia

**DOI:** 10.3390/clinpract11020051

**Published:** 2021-06-11

**Authors:** Iveta Racko, Liene Smane, Lizete Klavina, Zanda Pucuka, Ieva Roge, Jana Pavare

**Affiliations:** 1 Department of Continuing Education, Children’s Clinical University Hospital, LV-1004 Riga, Latvia; liene.smane@rsu.lv (L.S.); lizete.klavina@bkus.lv (L.K.); zanda.pucuka@rsu.lv (Z.P.); ieva.roge@bkus.lv (I.R.); jana.pavare@rsu.lv (J.P.); 2 Department of Paediatrics, Riga Stradins University, LV-1007 Riga, Latvia

**Keywords:** COVID-19, SARS-CoV-2, multisystem inflammatory syndrome in children (MIS-C)

## Abstract

The total number of COVID-19 positive cases in Latvia has escalated rapidly since October 2020, peaking in late December 2020 and early January 2021. Children generally develop COVID-19 more mildly than adults; however, it can be complicated by multisystem inflammatory syndrome in children (MIS-C). This case study aims were to assess demographic characteristics and the underlying medical conditions, and clinical, investigative and treatment data among 13 MIS-C patients using electronic medical records. All 13 had acute illness or contact with someone who was COVID-19 positive two to six weeks before MIS-C onset. Only five of the 13 were symptomatic during the acute COVID-19 phase. The median age was 8.8 years; 11/13 patients were male, 10/13 had been previously healthy, and all 13 patients tested positive for SARS-CoV-2 by RT-PCR or antibody testing. The most commonly involved organ systems were the gastrointestinal (13/13), hematologic (13/13), cardiovascular (13/13), skin and mucosa (13/13), and respiratory (12/13) ones. The median hospital stay was 13 (interquartile range, 11 to 18) days; 7/13 patients received intensive care, 6/13 oxygen support, and 5/13 received inotropic support. No deaths occurred. During the current pandemic, every child with a fever should have a clearly defined epidemiological history of COVID-19, a careful clinical assessment of possible multiple organ-system involvement, with a special focus on children with severe abdominal pain and/or skin and mucocutaneous lesions.

## 1. Introduction

Following the World Health Organization’s announcement on 11 March 2020 that the spread of the novel coronavirus (COVID-19) caused by severe acute respiratory syndrome coronavirus 2 (SARS-CoV-2) had reached the scale of a global pandemic, the Latvian government announced a national state of emergency on 12 March to slow the spread of the disease in the country. In 2020, Latvia had a total population of 1.908 million and by March 2021 SARS-CoV-2 infection had been confirmed in 4.8% of the total population (90,997 individuals), of whom only 8.7% (7883) were children under 19 years of age [1]. Most of the children had a mild or moderate course of the disease and were treated at home, but 83 patients had more severe manifestations, necessitating hospitalization at Riga Children’s Clinical University Hospital. The total number of COVID-19 positive cases has progressed rapidly since October 2020, peaking in late December 2020 and early January 2021 [2]. During this peak of the infection, the first cases of MIS-C were diagnosed at Riga Children’s Clinical University Hospital.

MIS-C is a rare complication that appears to be linked to COVID-19 and develops as a result of an, as yet, unspecified immune dysregulation with an excessive inflammatory response [3,4,5]. Several reports have suggested that children could still develop MIS-C despite an asymptomatic and mild course of SARS-CoV-2 [6,7]. The definition of MIS-C is based on the following principal elements: age, presence of fever, increased levels of inflammatory markers, the involvement of more than two organ systems, temporal relation to COVID-19 infection or exposure, and exclusion of other diagnoses [8]. We describe the features of epidemiology, clinical and investigative data, and treatment management in detail for 13 MIS-C patients admitted to Riga Children’s Clinical University Hospital. All 13 had documented fever >38.0 °C for ≥24 h at the time of presentation, severe illness involving more than two organ systems, and laboratory evidence of inflammation; equally, they all were linked to SARS-CoV-2 infection.

## 2. Materials and Methods

The present study is a case series that includes all patients with a temporal relation to SARS-CoV-2 infection and other main diagnostic criteria of MIS-C published by the US Centers for Disease Control and Prevention (CDC) [9]. Between 20 December 2020 and 19 February 2021, a total of 13 children aged from 4 to 17 years with MIS-C were admitted to the Children’s Clinical University Hospital in Riga. Using the electronic medical records of these patients, demographic characteristics and underlying medical conditions, clinical, investigative, and treatment data were collected. This study was reviewed and approved by the ethics committee of Riga Stradins University and by the Institutional Review Board of the Children’s Clinical University Hospital (No. 6-1/07/35). We obtained informed consent from all the children’s parents or guardians.

## 3. Results

### 3.1. Case Reports

#### 3.1.1. Demographic Characteristics and Underlying Medical Conditions

MIS-C symptoms developed two to six weeks after acute illness or contact with a COVID-19 positive person in all 13 patients, and they were hospitalized on the third to seventh day of illness (median five, interquartile range (IQR) four to six days). Only five patients out of the 13 were symptomatic during the acute COVID-19 phase; four had mild symptoms (subfebrility, cough, anosmia, and loss of taste), while one had a severe course of the disease and was admitted to hospital before the onset of MIS-C. The 13 children ranged in age from 4 to 17 (median age 8.8), 11/13 were boys and all them came from different parts of the country. All patients were European. Overall, 10/13 patients had no reported underlying medical conditions; one had bronchial asthma, another had Autism Spectrum Disorder (ASD), while another had gallstone disease. The patient with ASD is the one who had a severe course of acute COVID-19.

#### 3.1.2. Initial Signs and Symptoms

All study patients had a documented fever >38.0 °C for ≥24 h at the time of presentation, and severe illness involving at least four organ systems. The most commonly involved organ systems were the gastrointestinal (13/13), hematologic (13/13), cardiovascular (13/13), skin and mucosa (13/13), and respiratory (12/13) ones.

**Figure 1:**
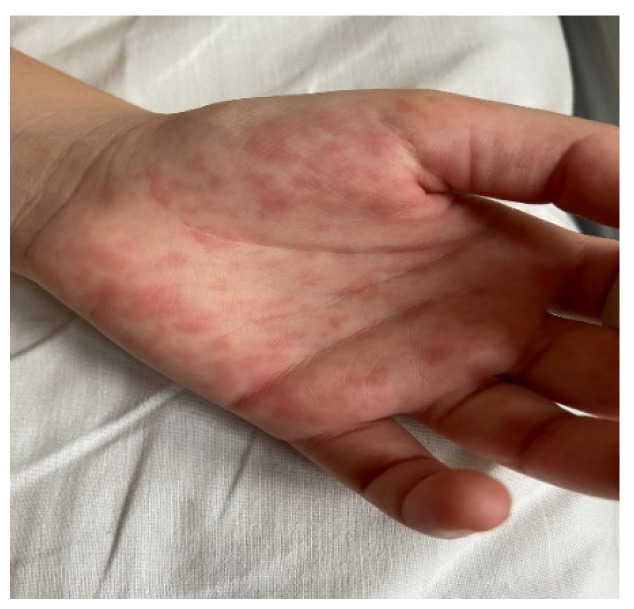
Maculopapular rash.

**Figure 2:**
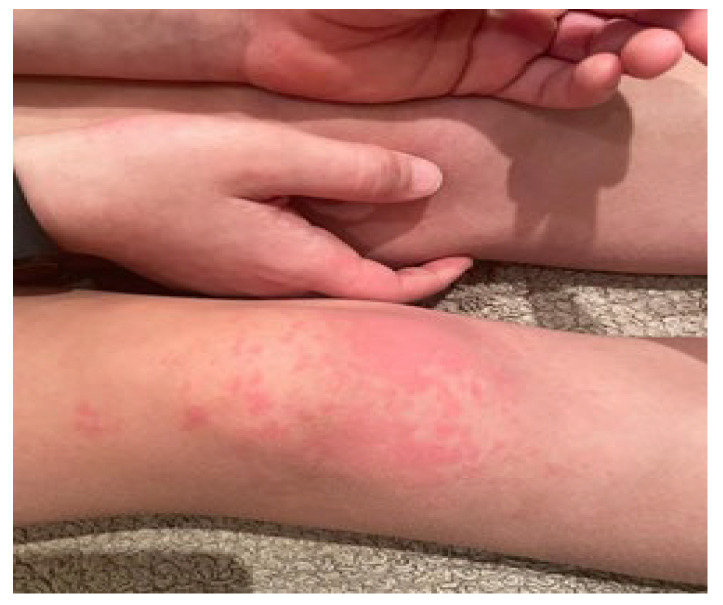
Maculopapular rash. Swelling and hyperemia of hands.

**Figure 3:**
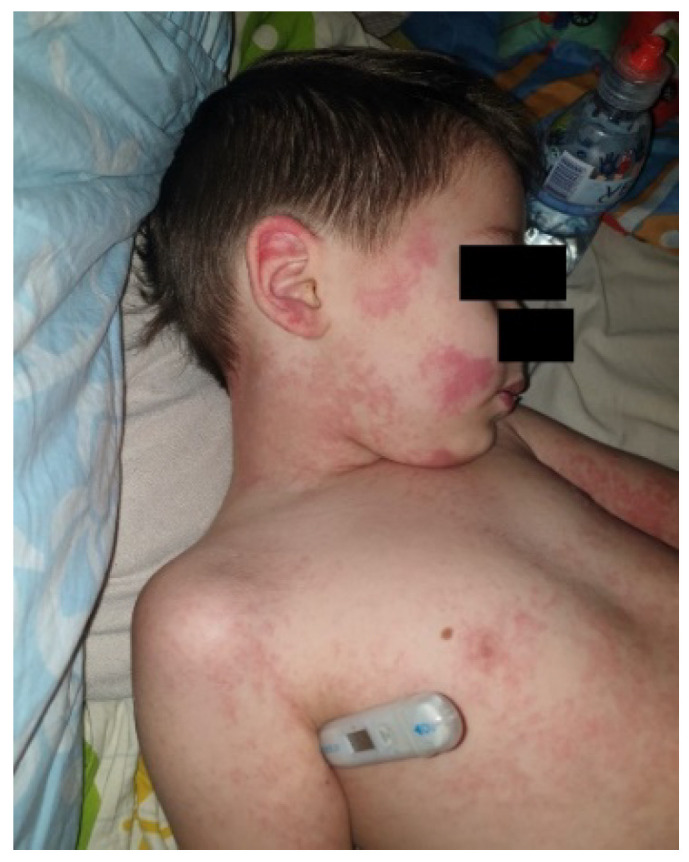
Maculopapular rash.

**Figure 4:**
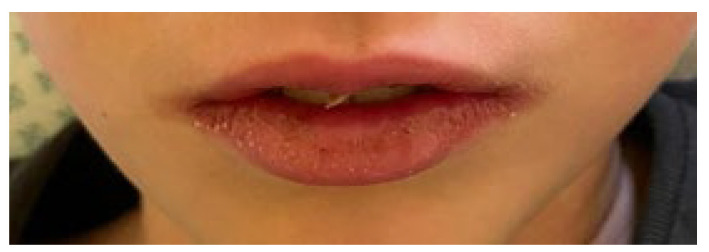
Cracking and hyperemia of lips.

**Figure 5:**
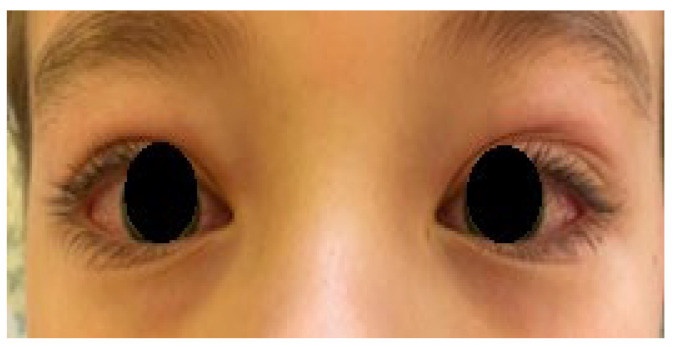
Conjunctival injection.

**Figure 6:**
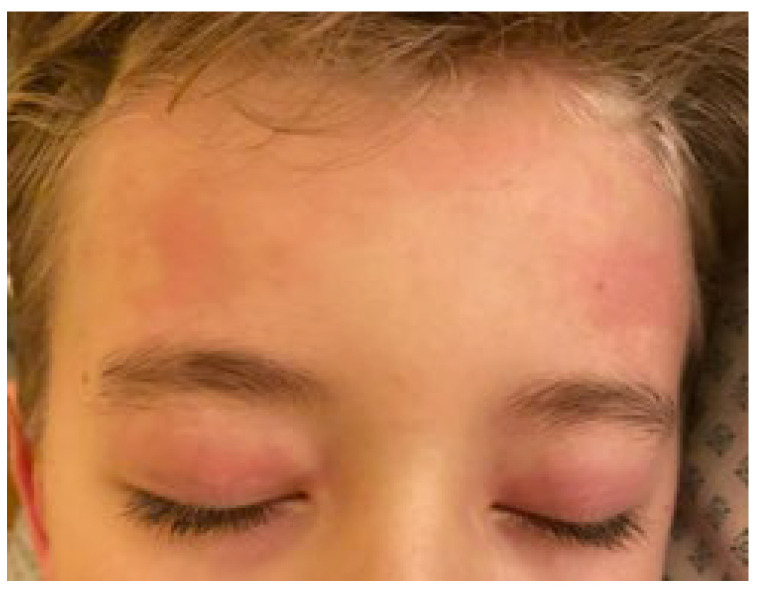
Periorbital edema and hyperemia of skin.

**Figure 7:**
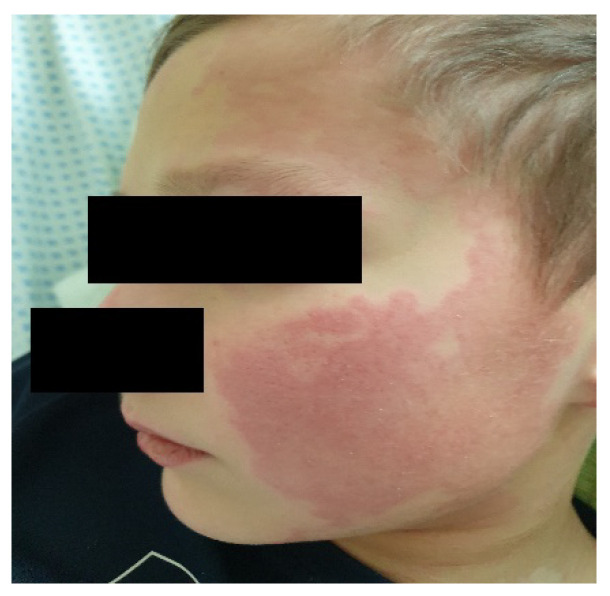
Cheek erythema.

The estimated median duration of fever was 7 (IQR 5.75–7.25) days. All patients exhibited skin and mucocutaneous lesions. Maculopapular rash (10/13) (Figure 1, Figure 2 and Figure 3), cracking and hyperemia of lips (12/13) (Figure 4), conjunctival injection (11/13) (Figure 5), swelling and hyperemia of hands and feet (9/13) (Figure 2), oral mucosal changes (9/13), and periorbital edema (8/13) (Figure 6) were among the most common findings. In addition, Figure 6 and Figure 7 show hyperemia of the skin. All 13 patients had acute gastrointestinal symptoms on admission, including abdominal pain (11/13), vomiting (9/13). and diarrhea (6/13). Two patients underwent diagnostic laparoscopy—one with an acute ileus and another with suspected acute appendicitis. Respiratory symptoms occurred in 12 patients overall, including a cough (10/13), acute respiratory distress with shortness of breath and tachypnea (6/13), desaturation episodes (3/13), and chest pain (2/13). Neurologic symptoms were present in (10/13) and acute cardiovascular manifestations in all 13 study patients. Five patients were hypotensive at the time of admission. Patients’ clinical characteristics are summarized in Table 1.

#### 3.1.3. Laboratory Markers and Additional Diagnostics

Initial laboratory criteria for strongly suspected MIS-C [9] as elevated C-reactive protein (CRP) ≥30 mg/L and/or erythrocyte sedimentation rate (ESR) >40 mm/h plus lymphopenia <1000 or thrombocytopenia <150 × 10^3^ or hyponatraemia <135 mmol/L were met in all 13 MIS-C patients. CRP was elevated in all 13 cases, median 187.01 (IQR 135.61–249.15) mg/L, elevated ESR in 11/13 patients, median 48 (IQR 40.5–65.5) mm/h, lymphopenia in 11/13 cases, median 550 (IQR 440–650) μL, thrombocytopenia in 12/13 cases, median 112 (IQR 96–134.25) μL, and hyponatremia in 8/13, median 129.65 (IQR 126.8–131.25) mmol/L.

Additional laboratory evidence of inflammation included increased serum ferritin with median 583.2 (IQR 511.6–861.1) ng/mL and serum cytokine interleukin 6 (IL-6) 194 (IQR 150–320) mg/L in all 13 cases. Lactate dehydrogenase (LDH) was elevated in 6/13 patients with a median 332 (IQR 325.75–342.25) μ/L. Hypoalbuminemia was observed in 13/13 cases, median value 26.17 (IQR 24.3–32.12) g/L. All 13 patients had increased coagulation marker D-dimer, median 5.97 (IQR 3.31–10.47) mg/L, and fibrinogen in 9/13, median 5.66 (IQR 4.81–7.01) g/L. Blood tests also revealed elevated levels of cardiac troponin I in 9/13 patients with a median of 93.4 (IQR 46.1–132.1) ng/mL and N-terminal pro B-type natriuretic peptide in all 13 cases with a median 7218 (IQR 2434–17134) pg/mL.

Pathological changes in echocardiography were observed in 10/13 patients; the most common findings included valvular insufficiency: mitral (6/13) and tricuspidal (8/13), decreased left ventricular ejection fraction (3/13). In electrocardiography (ECG), various changes of ST-segment or T-wave (11/13), bradyarrhythmias (6/13), tachyarrhythmias (6/13), intraventricular conduction defects (8/13) were observed. Three patients out of all 13 had signs of myocarditis by elevated cardiac biomarkers in conjunction with clinical signs and ECG and echocardiographic findings, and one of them was diagnosed with myocarditis by cardiac MRI. Altered chest radiographs were found in 10/13 patients. Pleural effusion was found in nine patients by ultrasonography. Table 2 shows all laboratory and radiographic findings in children with MIS-C.

#### 3.1.4. Link to SARS-CoV-2

All study patients were linked to SARS-CoV-2 by having been in contact with COVID-19-positive people two to six weeks before MIS-C symptoms developed. Only five patients out of the 13 were symptomatic during the acute COVID-19 phase, and three of them were tested for SARS-CoV-2 by PCR at that time–two were found to be positive. For the other 11 patients, SARS-CoV-2 RNA testing was completed at the time of MIS-C admission, and of these one patient had positive SARS-CoV-2 RNA by rapid molecular testing, but was negative by PCR. All 13 patients had a positive serology for SARS-CoV-2.

#### 3.1.5. Treatment and Clinical Course

Seven patients of all 13 required admission to the pediatric intensive care unit (PICU) for a median two (IQR 1.25–2.75) days’ stay because of hemodynamic instability, of whom five patients required inotropic support with epinephrine or norepinephrine, in one case hypotension was corrected with a bolus of saline. Oxygen support was required in six patients due to respiratory distress or desaturation, but mechanical ventilation was not needed.

Antimicrobials were prescribed for all the patients treated in the hospital. All received intravenous immunoglobulins (IVIG), glucocorticosteroids and acetylsalicylic acid (AAS) according to the indications. Two children received methylprednisolone pulse therapy. Anticoagulation therapy was completed in eight patients, one of whom received the treatment dose. Additionally, nine patients received diuretics and one patient received an interleukin-1Ra (Anakinra).

The median length of hospitalization was 13 (IQR 11–16.5) days for PICU patients and 15 (IQR 11.25–19.5) days for children treated on the hospital ward. There were no deaths among this group of patients.

## 4. Discussion

This report describes our first clinical experience of Latvian patients with MIS-C. In our study MIS-C cases occurred two to six weeks after acute illness or contact with a COVID-19 positive person after the COVID-19 infection peaked in Latvia. This was similar to previous studies reported in Europe [10,11,12,13]. We observed a tendency for children to be hospitalized at a relatively late stage in their illness, as observed in Santiago [14]. Moreover, in the case studies presented here, the children were most frequently treated in an outpatient setting with antibiotics used to treat other suspected diseases, including scarlet fever, gastrointestinal infections or acute appendicitis. Neither the children’s parents nor the outpatient doctors associated these conditions as a sequele after the COVID-19, perhaps, due to the mild or even asymptomatic course of the disease in children. [15]. This is also the reason why diagnostic tests were performed less frequently following exposure to COVID-19, although symptoms were displayed after that. Since PCR and anti-SARS-CoV-2 antibodies can often be negative, the careful acquisition of an epidemiological history is essential. To date, studies have indicated that males may be overrepresented [16,17]. In our study, 11/13 were boys and, overall, the majority of patients were previously healthy.

In our series, the median age was 8.8 years. Rafferty et al., based on the available studies, reported that the median age of children who developed MIS-C varied from seven to 10 years [3]. The most common clinical presentation was persistent fever along with dermatological, mucocutaneous and gastrointestinal features, similar to other reports [10,12,18]. Given that 100% of our study patients had acute gastrointestinal symptoms and all of them had multi-organ involvement, it has become essential to assess further the involvement of other organ systems in all children with a fever, and especially those with severe abdominal pain. For example, Belhadjer et al. reported gastrointestinal involvement in more than 80% of patients [19]. Gastrointestinal manifestations in MIS-C can present in a similar way to many other infectious diseases in children. In our study, abdominal pain was the most common gastrointestinal symptom. Two patients needed surgery because of this initial suspicion of acute abdominal symptoms. In the Jackson report, which described one clinical case, abdominal pain also mimicked acute appendicitis [20]. In fact, all our patients had skin and mucocutaneous involvement. Dermatological manifestations are the top clinical manifestations in children with MIS-C, as mentioned in other studies [16,21].

In the current study, we report that 100% of patients had cardiac involvement. The systematic review by Abrams et al. noted that 71% of MIS-C cases had cardiovascular symptoms [22]. These findings suggest that patients with MIS-C should always be closely monitored for cardiovascular function, since the majority of them have severe manifestations including shock, hypotension, arrhythmias and myocarditis. Nevertheless, it is also important to monitor arterial blood pressure and other possible signs of shock requiring immediate treatment. Patients with multi-organ system involvement require medical care in a tertiary-level hospital from a multidisciplinary team.

Laboratory evidence of systemic inflammation, myocardial dysfunction and coagulation activation has been consistently documented in previous reports [21,23,24]. All children in the present study had positive initial laboratory criteria for strongly suspected MIS-C, such as elevated CRP and/or ESR plus lymphopenia or thrombocytopenia or hyponatremia [25]. Given that they are easy to perform, these analyses are recommended as additional screening tools that can be used in an outpatient setting or a regional hospital.

There are known few clinical practice recomendations regarding treatment for MIS-C by several organisations [8,9,26,27]. Ramcharan et al. reported favorable outcomes in treatment plans with IVIG and corticosteroids [21]. Similarly, our experience showed good outcomes from using IVIG, corticosteroids, AAS and anticoagulants.

Hospitalization was longer due to the general condition of the children. Seven patients of all 13 required admission to the PICU. Similar to our study, Tolunay et al. also reported median duration of hospitalization 12,5 days in an article “Multisystem Inflammatory Syndrome in Children (MIS-C) Associated with COVID-19: A case series experience in a Tertiary Care Hospital of Southern Turkey [28].

Finally, this study has the limitation of representing a small case group. Only 13 children were enrolled. Collaboration is needed at national and international levels. Thus, a larger sample study should be used to confirm these results.

## 5. Conclusions

In the current pandemic, where the incidence of COVD-19 has peaked, every child with a fever should have a well-defined epidemiological history, and a careful clinical assessment of possible multiple organ-system involvement, with a special focus on those with severe abdominal pain and/or skin or mucocutaneous lesions. Before hospitalization or transfer to a university hospital, vital signs should be carefully monitored, intravenous rehydration and antibacterial therapy should be provided, and initial laboratory tests should be performed. Any signs of shock should be assessed and treated immediately. Both members of the public and medical staff need to be further educated about the possible late manifestations of COVID-19.

## Figures and Tables

**Table 1:** Demographic and clinical characteristics of all 13 patients with MIS-C.

Characteristic	Total
Sex, *n*: Female: Male:	2/13 11/13
Age in years, range, median	4–17, 8.8
Comorbidities, *n*	3/13
Days in hospital, median (IQR)	13 (11–18)
Outcome	Recovery
PICU admission, *n* Days in PICU, median (IQR)	7/13 2 (1.25–2.75)
Clinical characteristic:	
Duration of symptoms at admission, median days (IQR)	5 (4–6)
Days with fever, median (IQR)	7 (5.75–7.25)
No. of organ systems involved, *n*: 2–3 4–5 ≥6	0/13 13/13 0/13
Clinical manifestations, *n*: Fever	13/13
Rash: - Maculopapular - Petechiae	10/13 2/13
Mucocutaneous lesions: - Cracking/hyperemia of lips - Strawberry tongue - Oropharyngeal erythema - Conjunctival injection	12/13 2/13 9/13 11/13
Extremity changes: - Swelling/hyperemia of hands and feet Cervical lymphadenopathy >1.5 cm D Gastrointestinal: - Abdominal pain - Ileus - Vomiting - Diarrhea	9/13 7/13 11/13 3/13 9/13 6/13
Cardiovascular: - Hypotension - Tachycardia - Myocarditis - Congestive heart failure - Cardiac dysfunction	5/13 13/13 1/13 2/13 10/13
Respiratory: - Cough - Shortness of breath, tachypnea - Desaturation - Chest pain - Pneumonia - Pleural effusion	10/13 6/13 3/13 2/13 6/13 8/13
Neurologic: - Headache - Dizziness - Meningism/photophobia - Hyperesthesia - Emotional lability - Unsteady gait	6/13 3/13 4/13 2/13 2/13 1/13
Other: - Periorbital edema - Skin peeling of hands and feet - Hepatomegaly - Splenomegaly	8/13 2/13 6/13 3/13

**Table 2:** Laboratory values and radiographic findings in the patients with MIS-C.

Characteristic	Results
	The Median of Peak Values (IQR)
Initial laboratory criteria, (reference ranges): - CRP, (0–5) mg/L - ESR, (0–15) mm/h - Lymphopenia, (0.97–4.28) (×10^3^ μL) - Thrombocytopenia, (175–369) (×10^3^ μL) - Hyponatremia, (132–145) mmol/L - Hypoalbuminemia, (38–54) g/L	187.01 (135.61–249.15) 48 (40.5–65.5) 0.55 (0.44–0.65) 112 (96–134.25) 129.65 (126.8–131.25) 26.17 (24.3–32.12)
Additional inflammatory markers: - IL-6, (0–2) pg/mL - Ferritin, (20–200) ng/mL - LDH, (120–300) U/L	194 (150–320) 583.2 (511.6–861.1) 332 (325.75–342.25)
Cardiac biomarkers: - NT-Pro BNP, (0–125) pg/mL - Troponin, (0–19) ng/mL	7218 (2434–17134) 93.4 (46.1–132.1)
Coagulation parameters: - D-dimer, (0–0.55) (mg/mL) - Fibrinogen, (1.7–4.2) (mg/dL)	5.97 (3.31–10.47) 5.66 (4.81–7.01)
	***n***
Chest X-ray: - Interstitial edema/thickening - Pleural effusion - Inflammation - Bronchial obstruction, drainage disorder - Enlarged heart	3/13 3/13 4/13 4/13 1/13
Electrocardiography: - Various changes of ST-segment or T-wave - Bradyarrhythmias - Tachyarrhythmias - Intraventricular conduction defects - AV dissociation	11/13 6/13 6/13 8/13 1/13
Echocardiography: - Valvular insufficiency: - mitral - tricuspidal - aortal - pulmonal - Pericardial effusion - Decreased LV ejection fraction - RV, RA dilatation	6/13 8/13 2/13 1/13 2/13 3/13 1/13
Abdominal ultrasonography: - Ascites - Hepato- and/or splenomegaly - Mesadenitis - Renal parenchymal changes, cystitis - Pericholecystitis - Effusion in the abdominal cavity/pelvis	2/13 4/13 3/13 1/13 1/13 4/13
Pleural ultrasonography-effusion:	9/13
Computed tomography of the lungs: - Bilateral polysegmental pneumonia - Fibrotic changes - Cardiac magnetic resonance-myocarditis	2/13 2/13 1/13
SARS-CoV-2 test results at the admission: - Positive nasopharyngeal RT-PCR - Positive serology - History of COVID-19 (+) contact	0/13 13/13 13/13

Abbreviations: CRP, C-reactive protein; ESR, Erythocyte sedimentation rate; IL-6, Interleukin 6; LDH, Lactate dehydrogenase; NT-Pro BNP, N-terminal pro b-type natriuretic peptide; AV, Atrioventricular; RA, Right Atrium; RV, Right Ventricular; RT-PCR, Reverse transcription polymerase chain reaction.

## Data Availability

The data presented in this study are available on request from the corresponding author. The data are not publicly available due to privacy issues.
